# Recent Advances in Prevention and Recovery in People with Schizophrenia and Related Disorders

**DOI:** 10.3390/bs14110988

**Published:** 2024-10-24

**Authors:** Ashley M. Schnakenberg Martin, Kelsey A. Bonfils

**Affiliations:** 1 Department of Psychiatry, Yale University School of Medicine, New Haven, CT 06511, USA; 2 Psychology Service, VA Connecticut Healthcare System, West Haven, CT 06516, USA; 3 School of Psychology, University of Southern Mississippi, Hattiesburg, MS 39406, USA; kelsey.bonfils@usm.edu

## 1. Introduction

People with schizophrenia-spectrum disorders often experience a combination of psychological symptoms and functional impacts, such as difficulty in social relationships, finding or maintaining employment, and attending school [1,2,3,4]. However, people with these disorders display remarkable resilience, and a large body of literature demonstrates that people can attain personal recovery from schizophrenia and work toward developing meaningful and fulfilling lives [5,6]. This Special Issue aims to contribute to our understanding of the determinants that lead to the recovery of people who have schizophrenia and related disorders (e.g., bipolar disorders or other psychotic illnesses) and the determinants of prevention or recovery for those considered to be at-risk for the development of psychosis (e.g., prodromal, clinical high risk, or high schizotypy). 

Recovery unfolds in a personally meaningful manner and may include both objective (e.g., return to work or school) and subjective (e.g., quality of life, perceived recovery) [7,8,9] indicators. We were lucky in this Special Issue to receive submissions examining recovery and its correlates across this spectrum of interrelated outcomes. Collectively, these articles showcase recent advances in our understanding of recovery, emphasizing the importance of the whole person, both in our investigations of recovery and within our treatment for those with schizophrenia and related disorders.

## 2. Determinants of Recovery

In the present issue, researchers investigate determinants of recovery from a wide range of perspectives, including speech, employment, introspective accuracy, and healthcare utilization. The work by Kilicoglu and colleagues found that language-derived indices of metacognitive function relate to key social outcomes. Metacognition can be broadly thought of as a set of abilities that enables one to think about what is happening in one own’s mind as well as in the minds of others [9]. Their study found that greater abilities to think about one’s own thoughts were associated with worse self-reported functioning, while the ability to think about others’ thoughts was associated with better aspects of performance-based social functioning [10]. Relatedly, Bettis and colleagues demonstrated that distinct themes of alienation, interpersonal tension, personal benchmarks, and adverse experiences may precede disorganized speech in people with schizophrenia, with implications for modifying psychotherapy based on a person’s unique risk factors and disorganization [11]. Employment hope has also been observed to play an important role in an individual’s perception of one’s own recovery, and thus should be considered in clinical efforts to enhance an individual’s recovery course [12].

Introspective accuracy, or one’s capacity to correctly estimate their own abilities, skills, and performance, is also described in this issue to be less intact in those with schizophrenia and schizoaffective disorder, compared to those with bipolar disorder [13]. Interestingly, there also appears to be a differential relationship between introspective accuracy and sleep quality across these diagnostic groups, suggesting a need for future work to understand what might drive diagnostic differences in these relationships and how those factors may be leveraged to promote recovery. Soreca and colleagues investigated how the COVID-19 pandemic influenced healthcare utilization by those with serious mental illnesses (SMI) in the Veterans Health Administration. They found that veterans with SMI had a decreased utilization of healthcare and an increased mortality as compared to veterans without SMI, which is best explained by pre-existing demographic and health factors—not necessarily a positive COVID-19 test result [14]. This important work highlights the importance of healthcare for those with SMI and the need to closely examine healthcare disparities experienced by this vulnerable group. 

Lastly, Harris and colleagues provide a thoughtful review of the use of subjective versus objective assessments of cognition and function in individuals with schizophrenia [15], both important areas with a high relevance to recovery. Notably, this work identified important cognitive and clinical features that impact self-assessment and underscores the need for a comprehensive assessment that integrates subjective and objective sources of data. 

## 3. Treatment of Schizophrenia-Spectrum Disorders

In the present issue, investigators also consider the development and advancement of evidence-based treatments for individuals with serious mental illness, such as Metacognitive Reflection and Insight Therapy (MERIT), an intervention aimed at improving insight and metacognition [16]. In a qualitative study of 27 individuals with serious mental illness who received MERIT as part of a randomized controlled trial [17], Tsuck Ram and colleagues demonstrated that satisfaction with MERIT was related to domains of self-experience (e.g., self-management, reflectiveness) [18]. Further, perceived changes were reported by clients as occurring related to therapeutic alliance, therapist interventions, and the individual being an active agent of change in their own course of recovery [18]. Similarly, in a second study evaluating a group format of MERIT (MERITg), Musket and colleagues demonstrated that group attendance was positively associated with improvements in self-reported recovery-oriented beliefs [19].

Finally, in a systematic review of 33 qualitative studies on individual psychotherapy for people with psychosis (including cognitive therapies, acceptance and mindfulness approaches, trauma therapies, metacognitive therapy, and music therapy) by Faith and colleagues, investigators sought to understand the client-reported benefits of psychotherapy, how psychotherapy impacts recovery, and the experience of the psychotherapy process [20]. They observed that across interventions, the therapeutic relationship was particularly important in the promotion of recovery [20]—consistent with findings reported by Tsuck Ram and colleagues [18], described above.

## 4. Understanding High Risk States to Contribute to Prevention and Recovery Efforts

In a large study (n = 897), Hammer and colleagues used network analysis to demonstrate that the network structure of the Interpersonal Reactivity Index, a commonly used measure of empathy (including in schizophrenia-spectrum populations), varies in high and low schizotypy groups [21]. This work has critical implications for future work investigating empathy, an important determinant of social relationships, across healthy, high-risk, and schizophrenia samples. Further, work by DeBats and colleagues investigated the relationship between schizotypy, social functioning, and the frequency and enjoyment of social activities [22]. The authors identified small relationships between social activities and social functioning, and further found that social activities were inversely associated with negative and disorganized schizotypal traits, with negative traits having the most robust association with both the frequency and enjoyment of social activities. This study offers insight into the complex relationships between schizotypy, social functioning, and the frequency and enjoyment of social activities, and opens doors to future research with the utilization of the authors’ frequency–enjoyment matrix in both high-risk and clinical populations. 

To better understand the high-risk state, Monnette and colleagues uniquely evaluated how racial discrimination and traumatic experiences relate to schizotypy in a large sample of young adults (n = 770) [23]. Importantly, investigators found that trauma was associated with positive, negative, and disorganized schizotypy and that racial discrimination was associated with positive and disorganized schizotypy. Findings suggest the need for policy and system-level change to mitigate the occurrence of racial discrimination and to decrease the disparities in psychosis risk.

In addition to examinations of risk and recovery in high risk samples, Leonhardt and colleagues outlined the potential for use of MERIT to address the specific needs of those at clinical high risk for psychosis [24]. The authors suggest that MERIT could provide unique benefits through its aims to improve agency and self-directed recovery by promoting metacognition. They provide careful consideration of how the elements of MERIT may serve this population as well as a clinical example of how such work may unfold. As a complement to this work, Wiesepape and colleagues review metacognition as a transdiagnostic determinant of recovery in individuals at risk for and with schizophrenia spectrum disorders [25]. In their review, the authors propose that individuals with high levels of schizotypy are likely to benefit from early metacognitively oriented interventions, such as MERIT or Evolutionary Systems Therapy for Schizotypy (ESTS), particularly in regard to deficits in metacognitive capacity and the ability to understand and reflect on one’s own mind and that of others. 

## 5. Conclusions

Taken together, these studies highlight recent scientific advances in the investigation of determinants of prevention and recovery in people with schizophrenia and related disorders. The present issue emphasizes (1) unique determinants of recovery for those with schizophrenia and related disorders, including one’s speech, employment hope, introspective accuracy, and treatment utilization; (2) recent advances in novel treatments for individuals with serious mental illness, including MERITg, and the importance of therapeutic alliance for recovery; and (3) important insights into high risk states relevant for prevention efforts, including studies on empathy, social activities, racial disparities in traumatic experience, and metacognition. 

In aggregate, the studies presented herein identify key areas for future research to investigate how to leverage these insights in larger studies in the prevention of psychosis and the support of self-directed recovery. Specifically, future research would benefit from additional, independent, randomized controlled and longitudinal trials on the proposed interventions for those at risk of psychosis to test the efficacy of interventions such as MERIT and MERITg. Future work should also consider important individual factors that may influence these relationships, such as biological sex and the impact of substance use, such as cannabis, given the rapidly increasing prevalence of cannabis use globally and in those with serious mental illness. Finally, future work would benefit from expanding to include multimodal approaches (e.g., EEG or other neurophysiological outcomes) to better understand the neural networks responsible for the observed clinical outcomes and responses to treatment.

This Special Issue brings together many perspectives, but ultimately the articles and their authors present robust examinations and commentary in support of the resiliency seen in those with schizophrenia and related disorders. Critically, the Special Issue also makes several suggestions of how both researchers and practitioners alike can both foster and support recovery for those with serious mental illness. 
